# Macrophage MMP10 Regulates TLR7-Mediated Tolerance

**DOI:** 10.3389/fimmu.2018.02817

**Published:** 2018-12-04

**Authors:** Maryam G. Rohani, Elizabeth Dimitrova, Andrew Beppu, Ying Wang, Caroline A. Jefferies, William C. Parks

**Affiliations:** Departments of Medicine and Biomedical Sciences, Cedars-Sinai Medical Center, Los Angeles, CA, United States

**Keywords:** macrophage, tolerance, toll-like receptor, metalloproteinase, skin immunity

## Abstract

Using an *in vivo* model of tolerance to TLR7-induced skin inflammation, we found a critical role for macrophage-derived MMP10 in mediating immune hypo-responsiveness. Cutaneous exposure to Imiquimod (IMQ), a TLR7 agonist, induced acute expression of pro-inflammatory factors (IL1β, IL6, CXCL1) and neutrophil influx equally in both wildtype and *Mmp10*^−/−^ mice. However, whereas subsequent exposure (11 and 12 days later) to IMQ led to marked abrogation of pro-inflammatory factor expression in wildtype mice, *Mmp10*^−/−^ mice responded similarly as they did to the first application. In addition, the second exposure led to increased expression of negative regulators of TLR signaling (TNFAIP3, IRAK3) and immunosuppressive cytokines (IL10, TGFβ1) in wildtype mice but not in *Mmp10*^−/−^ mice. *In vitro* studies demonstrated that prior exposure of IMQ to bone marrow-derived macrophages (BMDM) made wildtype cells refractory to subsequent stimulation but did not for *Mmp10*^−/−^ macrophages. These findings expand the critical roles MMP10 plays in controlling macrophage activation to indicate that the development of immune tolerance to TLR7 ligand is dependent on this macrophage-derived proteinase.

## Introduction

Toll-like receptors (TLRs) are a family of highly conserved Pattern Recognition Receptors that are activated by pathogen-associated and damage-associated molecular patterns. In addition, TLRs contribute to dampening immune responses, which is beneficial in the resolution of inflammation and barring development of autoimmune diseases (1, 2). TLR7, an endosomal receptor that recognizes single-stranded RNAs from viruses or dying cells (3), has a critical role in the induction and modulation of autoimmunity, as in systemic lupus erythematosus (4).

Depending on stimuli within their microenvironment, resident, and infiltrated macrophages can differentiated into pro- or anti-inflammatory cells, referred to as M1 (classically activated) or M2 (alternatively activated) cells, respectively (5–7). Several proteins influence macrophage behavior, including some members of matrix metalloproteinase (MMP) family (8–11). For example, studies by our laboratory demonstrated that MMP28 and TIMP3 mitigate the pro-inflammatory activity of macrophages in response to infection and sterile injury (8, 9), and MMP8 enhances anti-inflammatory function of macrophages by increasing the bioavailability of TGFβ1 (10). We reported that stromelysin-2 (MMP-10), which is expressed by macrophages, promotes the activation of immunosuppressive and matrix-degrading programs in macrophages, including resident skin macrophages (12–14).

TLRs affect macrophage activation typically by promoting their polarization toward pro-inflammatory states (15). Indeed, macrophages deficient in TLR3, 7, 8, 9, or 13 have impaired pro-inflammatory responses to infection (16). Repeated stimulation of TLR7 with synthetic agonists induces tolerance and restrains inflammation in autoimmune and tumor models (17, 18). As macrophages are involved in immune tolerance (19, 20), including tolerance to TLR7 ligands (21, 22), we explored if MMP10 impacts macrophage responses to TLR7 activation. Our findings demonstrate that prior topical treatment with Imiquimod (IMQ), a TLR7 agonist, dampened proinflammatory responses, and promoted tolerance to subsequent application of IMQ at a different site. Using both *in vivo* and cell-based approaches, we found that these effects were dependent on MMP10 in macrophages. These observations expand the roles for MMP10 in being a critical effector of macrophage activation.

## Materials and Methods

### Animals

Age-matched (8–10 weeks) *Mmp10*^−/−^ mice (23) and wildtype littermates (C57BL/6J), near equal number of females and males, were used for these studies. All procedures were approved by the Institutional Animal Care and Use Committee at Cedars-Sinai Medical Center.

### Model of Immune Tolerance

**Figure 1:**
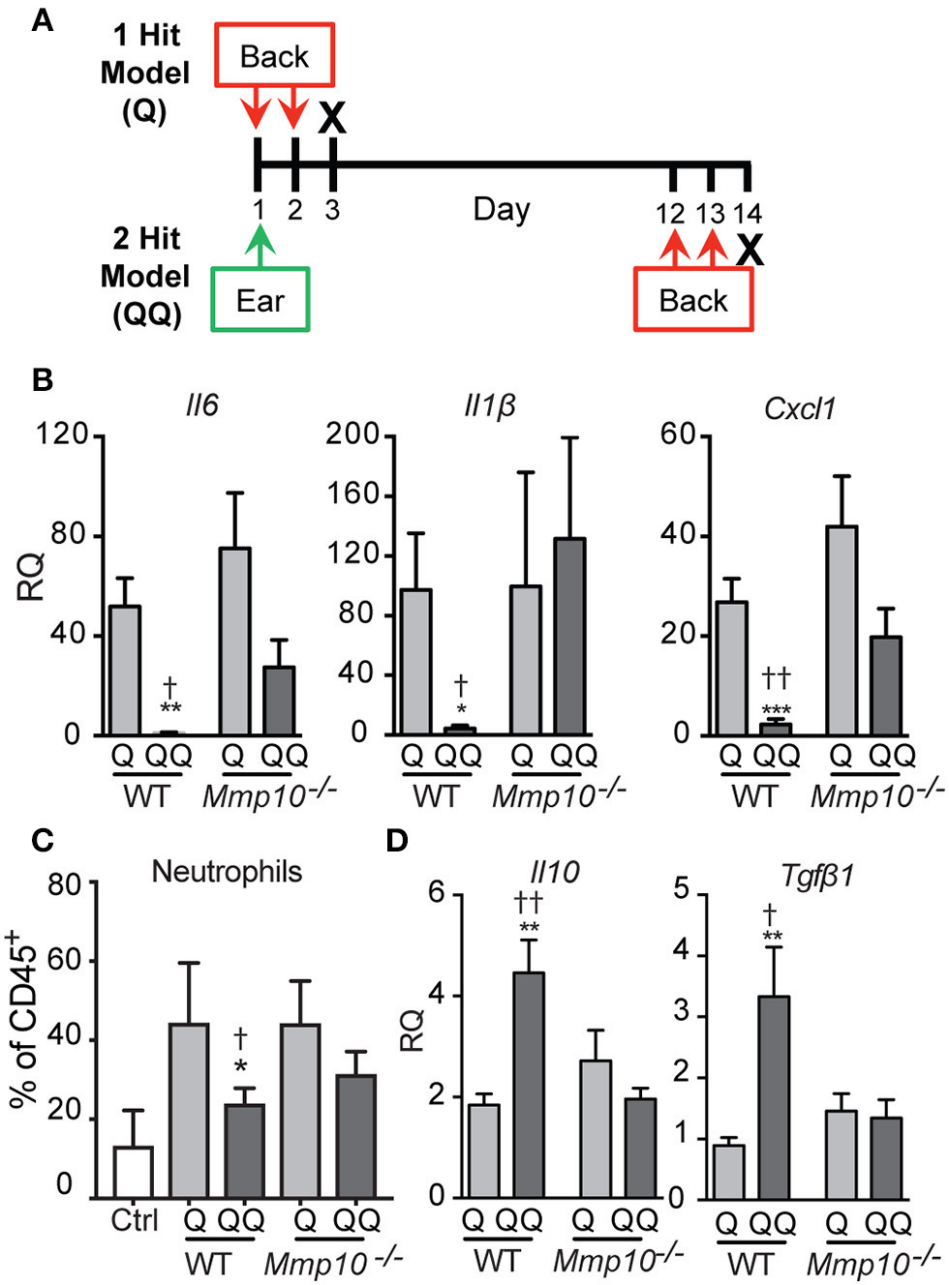
Tolerance to IMQ is dependent on MMP10. **(A)** Schematic diagram of *in vivo* tolerization protocol. **(B,D)** Back skin from wildtype and *Mmp10*^−/−^ mice treated with 1- or 2-hit model was homogenized and total RNA was isolated. Expression of target mRNAs was measured using qPCR and normalized to untreated (control) wildtype mice, *n* = 7–10 mice per group from two independent experiments. **(C)** Single cells isolated from back skin tissue were stained for CD45 and Ly6G. The frequency of Ly6G^+^ cells (a marker of neutrophils) on pre-gated CD45^+^ cells was assessed by flow cytometry. (*p* ≤ 0.05: ^*^WT-Q vs. WT-QQ and *Mmp10*^−/−^-Q vs. *Mmp10*^−/−^-QQ;WT-Q vs *Mmp10*^−/−^-Q and WT-QQ vs. *Mmp10*^−/−^-QQ; ^**^p ≤ 0.01, ^***^p ≤ 0.001,*p* ≤ 0.01).

We developed a 2-hit model (QQ; Figure 1A) to induce tolerance to TLR7 signaling. For this, the right ears of mildly anesthetized mice were treated with 25 mg Aldara cream (5% IMQ; Taro Pharmaceutical, Hawthorne, NY). Eleven (11) days later, the back was shaved, and 62.5 mg IMQ was applied on 2 consecutive days. For the 1-hit model (Q), mice received only the two consecutive back treatments (Figure 1A). Mice with mock treatment (shaved, no IMQ) served as controls. One day after the last application (day 3 for Q; day 14 for QQ) mice were sacrificed, and skin at the site of the second application and the inguinal and axillary lymph nodes were collected (Figure 1A).

### Assays

Total RNA was isolated from homogenized skin and cultured macrophages, and transcripts were assessed by quantitative real-time PCR (qPCR) as described (24). For flow cytometry, skin samples and lymph nodes were processed to obtain single-cell suspensions (13). Cell surface receptors were stained with conjugated antibodies to CD45 (30-F11), B220 (RA3-6B2), CD4 (GK1.5), CD8a (53-7.3), Ly6G (1A8), CD11b (M1/70), F4/80 (BM8), CD11c (N418), and MHC II (M5/114-15.2; eBioscience, San Diego, CA) and analyzed using a BD LSRFortessa Cytometer and FlowJo software (Tree Star, Ashland, OR). IL10 protein levels were measured using the Mouse IL10 ELISA Ready-SET-Go! Kit (eBioscience).

### Macrophage Studies

Isolation, culture, and activation of bone marrow-derived macrophages (BMDM) were done as described (25). Briefly, marrow cells were differentiated to macrophages by culturing in CSF-1-containing medium for 7 or 8 days. For TLR7 activation, BMDM were stimulated with IMQ (InvivoGen, San Diego, CA) in a 1- or 2-hit model. For the 2-hit model, BMDM (4 × 10^5^ in 12-well plate) were stimulated with 400 ng/ml IMQ in PBS for 4 h, washed, incubated in fresh medium for 18 h, then re-stimulated with 1 μg/ml IMQ for 4 (RNA isolation) or 16 h (protein analysis) before harvest of cells and media. For the 1-hit model, BMDM were treated with 1 μg/ml IMQ for 4 or 16 h, and then harvested. For adoptive transfer, recipient mice received 7 × 10^6^ wildtype GFP^+^ BMDM in 200 μl PBS via retro-orbital injection as described (13).

### Statistics

Statistical analyses were performed using Prism 5 (GraphPad software, LaJolla, CA). Data are presented as mean ± SEM. Statistical significance was determined using *t*-test. A *p*-value of ≤ 0.05 was considered statistically significant. In the figures, we used ^*^ to denote a significant difference between the 1-hit and 2-hit regimens in both wildtype and *Mmp10*^−/−^ mice and † to denote significant differences between wildtype and *Mmp10*^−/−^ mice.

## Results

### Model

As described under Methods, we used a 2-hit model (QQ; Figure 1A) to induce tolerance to TLR7 signaling. IMQ was applied to the right ears of mice, and 11 days later, IMQ was re-applied twice to the back. For the 1-hit model (Q), mice received only the two consecutive treatments (Figure 1A). One day after the last application, skin at the site of the second application together and lymph nodes were harvested. To track morbidity, we monitored weight loss in response to the 1-hit and 2-hit IMQ treatments. We observed similar weight loss 1 day after the last back treatment in both 1-hit and 2-hit model in both genotypes (Figure S1).

### MMP10 Moderates Pro-Inflammatory Responses Induced by TLR7 Ligation

To test the immune response to 1-hit IMQ application, we analyzed expression of *Il6, Il1b*, and *Cxcl1*, pro-inflammatory factors stimulated by TLR activation (26, 27), neutrophil influx, and expression of *ll10* and *Tgfb1*, two key immunosuppressive cytokines. As we found in other studies and tissues (12–14), in intact skin the expression of the cytokines studied here—except for TGFβ1 (average Ct ~29)–is low (average Ct >33), and the levels do not differ between wildtype and *Mmp10*^−/−^ mice (12–14). Similarly, the numbers of circulating and tissue leukocytes does not differ between naïve mice of either genotype (12–14).

In response to the acute 1-hit model, in which IMQ is applied on two consecutive days to an exposed area of back skin (Figure 1A), expression of *Il6* increased about 50-75-fold, *Il1b* about 100-fold, and *Cxcl1* about 25-40-fold, with no significant differences between wildtype and *Mmp10*^−/−^ mice (Figure 1B). Consistent with the expression of *Cxcl1*—a potent neutrophil chemoattractant—we found significantly more neutrophils in the back skin of both wildtype and *Mmp10*^−/−^ mice in response to the 1-hit model (Q, Figure 1C). Expression of *Tgfb1* was not stimulated by the 1-hit exposure and *ll10* increased slightly (~2-fold; Figure 1D). These findings indicate that MMP10 does not influence the initial response to TLR7 activation.

In contrast, we saw significant differences between wildtype and *Mmp10*^−/−^ mice in response to the 2-hit (QQ) model. For this model, mice are sensitized with a single topical application of IMQ to the ear then challenged 11 days later with consecutive applications of IMQ to flank skin (Figure 1A). Whereas expression of *Il6, Il1b*, and *Cxcl1* were close to basal levels in wildtype skin in response to the 2-hit model, these pro-inflammatory factors were all stimulated in *Mmp10*^−/−^ mice at the site of the second exposure, reaching levels comparable to those seen with the 1-hit (Figure 1B; QQ). In addition, whereas the neutrophil response was blunted in response to the 2-hot model in wildtype skin, their influx was stimulated in *Mmp10*^−/−^ mice (Figure 1B; QQ). Furthermore, whereas expression of *ll10* and *Tgfb1* increased in wildtype mice treated with the 2-hit model, expression of neither gene increased in *Mmp10*^−/−^ mice (Figure 1D). These findings suggest that development of immune tolerance to TLR7 ligation was dependent on MMP10.

### MMP10 Mediates Expression of Negative Regulators of TLR

**Figure 2:**
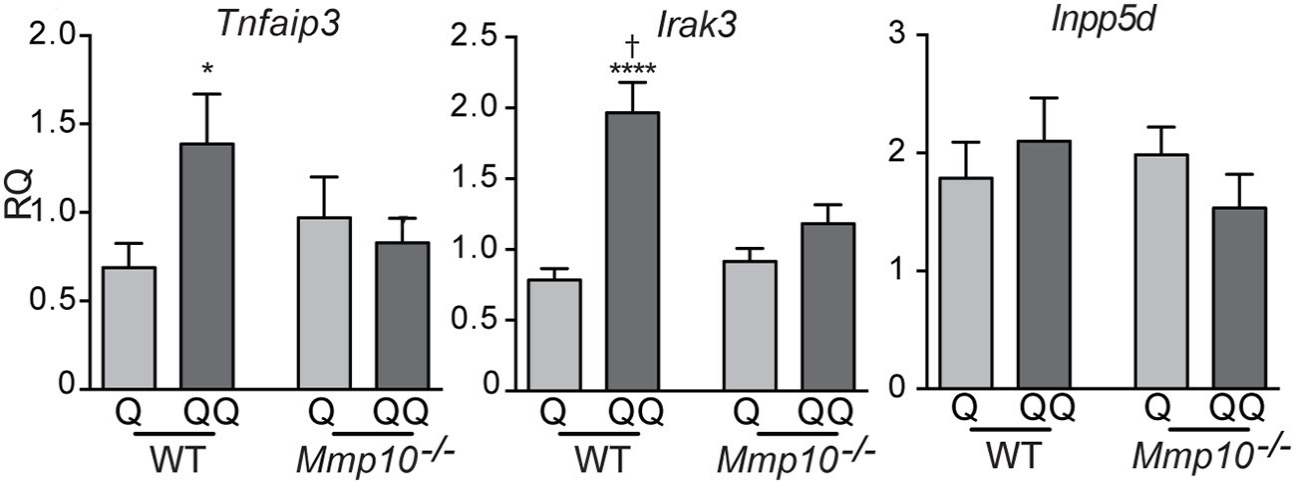
MMP10 promotes expression of negative regulators of Tlr7 signaling. RNA was isolated from the back skin from untreated (control) mice and wildtype and *Mmp10*^−/−^ mice treated with the 1-hit or 2-hit model. Levels of mRNAs for *Tnfaip3, Irak3*, and *Inpp5d* were measured by qPCR and normalized to *Hprt* and *Gapdh, n* = 7–10 mice per group from two independent experiments. Data are presented as fold increase compared to untreated wildtype control mice. (*p* ≤ 0.05: ^*^WT-Q vs. WT-QQ and *Mmp10*^−/−^-Q vs. *Mmp10*^−/−^-QQ;WT-Q vs. *Mmp10*^−/−^-Q and WT-QQ vs *Mmp10*^−/−^-QQ; ^****^p ≤ 0.0001).

To determine if the hypo-responsiveness we observed in the 2-hit model was due to tolerance to TLR signaling, we assessed expression of negative regulators of TLR signaling: *Tnfaip3, Irak3*, and *Inpp5d* (*Ship1*) (26, 28). Indicative of immune tolerance, expression of *Tnfaip3* and *Irak3* were elevated in the 2-hit model in wildtype mice (Figure 2). However, expression of these critical tolerance factors was not altered in *Mmp10*^−/−^ mice in response to the 2-hit model. Expression of *Inpp5d* did not differ between the 1- and 2-hit models and genotypes. Similarly, we observed increased levels of TNFAIP3 protein in the 2-hit model compared to the 1-hit model (Figure S2). These data indicate that MMP10 promotes tolerance by regulating expression of *Tnfaip3* and *Irak3*.

### MMP10 Regulates Immune Responses by Modulation of Macrophage Activation

**Figure 3:**
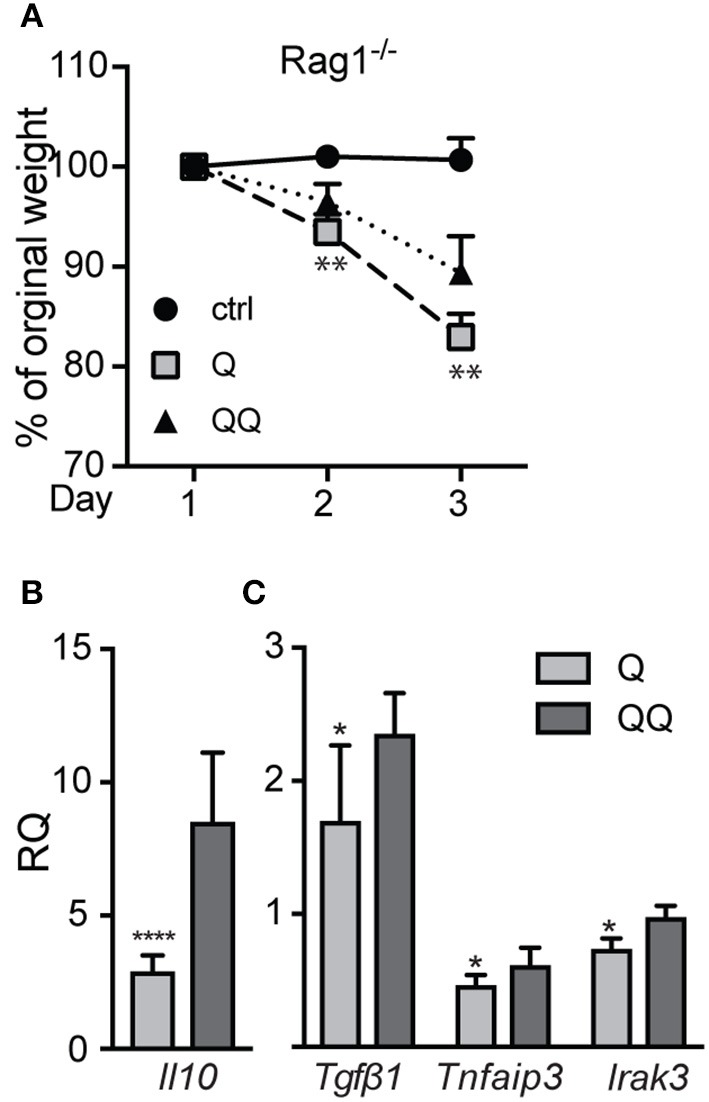
*Rag1*^−/−^ mice develop tolerance to TLR7 ligation. *Rag1*^−/−^mice were untreated (control) or treated with the 1- or 2-hit model. **(A)** Weight loss is shown as percent of the original weight (*n* = 8/group from two independent experiments). **(B,C)** RNA from back skin was used to measure expression of immune markers using qPCR. Data are normalized to *Hprt* and *Gapdh* and shown as fold changed relative to untreated controls. (^*^p ≤ 0.05 Q vs. QQ; ^**^p ≤ 0.01, ^****^p ≤ 0.0001).

To determine which cell types mediate the tolerance response, we investigated the possible role of lymphocytes. Although we found no differences in the number of B (B220^+^) and T lymphocytes (CD4^+^, CD8^+^) in lymph nodes between the 1- and 2-hit models in wildtype mice (Figure S3A) or in 2-hit treatment between wildtype and *Mmp10*^−/−^ mice (Figure S3B), their activation state could be shaped by other effector cells. To test if lymphocytes played a role in TLR7 tolerance, we treated *Rag1*^−/−^ mice, which lack mature T and B lymphocytes, with the two models. Following the 1-hit exposure (Q), *Rag1*^−/−^ lost about 20% body weight over the next 48 h, due to the systemic inflammation caused by cream application (29). However, following the 2nd treatment (QQ), weight loss in *Rag1*^−/−^ mice was significant less severe (Figure 3A). In addition, expression of *Il10* (30) were highly expressed in back skin of *Rag1*^−/−^ mice in response to the 2-hit model but not in 1-hit treatment (Figure 3B). We also found a significant increase in expression of *Tgf*β*1, TNFAIP3 and IRAK3*, although the differences were not as robust as for *Il10* (Figure 3C). Although we observed a trend for lower expression of pro-inflammatory markers *Il6, Il1b*, and *Cxcl1* between the 2-hit vs. 1-hit treatments (Figure S4), the differences were not statistically significant. These findings indicate that the development of negative regulation of immune responses to TLR7 ligands does not require B and T cells.

**Figure 4:**
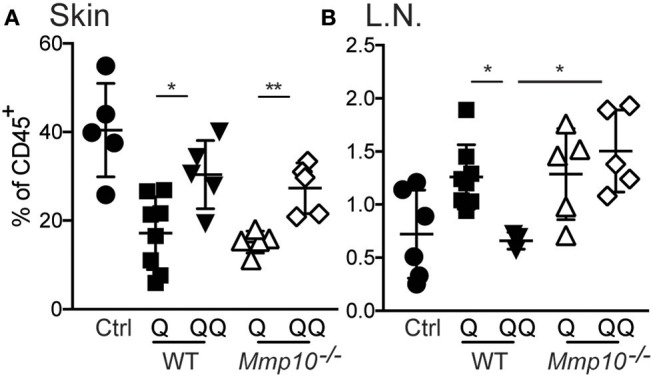
MMP10 does not influence influx of macrophages to the site of inflammation but does impact their efflux to lymph nodes. Back skin **(A)** and lymph nodes **(B)** samples were isolated from IMQ treated and untreated (control) mice. Single cells were isolated and stained with antibodies against CD45, Ly6G, CD11b, F4/80, CD11c, and MHC II. CD45^+^Ly6G^−^ cells were analyzed for expression of CD11b^+^, F4/80^+^ (macrophages) by flow cytometry. Each symbol represents data from one mouse, and data were collected from two separate experiments. (^*^p ≤ 0.05, ^**^p ≤ 0.01).

To test the role of myeloid cells, we isolated myeloid cells from back skin on day 12 after the initial ear treatment (thus, no second exposure to back skin), and we found—not unexpectedly—no difference in the numbers of neutrophils, macrophages, or dendritic cells between genotypes (Figure S5). Compared to untreated control group we observed significant decrease in population of macrophages in 1-hit treatment group (both in wildtype and *Mmp10*^−/−^ mice; Figure 4). In response to the second exposure of IMQ to back skin (QQ), the numbers of macrophages in both wildtype and *Mmp10*^−/−^ at the site of administration were significantly elevated compared to mice treated with just the 1-hit regimen (Figure 4A). We isolated skin draining lymph nodes and found a similar increase in the numbers of macrophages in response to the 1st IMQ exposure (Q) in both wildtype and *Mmp10*^−/−^ mice (Figure 4B), suggesting efflux of these cells from the skin at the site of exposure. However, in response to the 2nd hit, we detected a significant reduction in macrophages recovered from wildtype lymph nodes compared to 1-hit numbers (Figure 4B). In contrast, macrophage numbers remained elevated in *Mmp10*^−/−^ lymph nodes after the 2nd hit. Dendritic cells numbers did not change among conditions or between genotypes (Figure S6). Similar to what we found in skin wounds (13), these data indicate that MMP10 does not influence macrophage influx to sites of cutaneous inflammation but moderates the efflux of macrophages from sites of inflammation to draining lymph nodes in tolerant condition.

### Adoptive Transfer of Macrophages Restores Tolerance in Wildtype Mice

**Figure 5:**
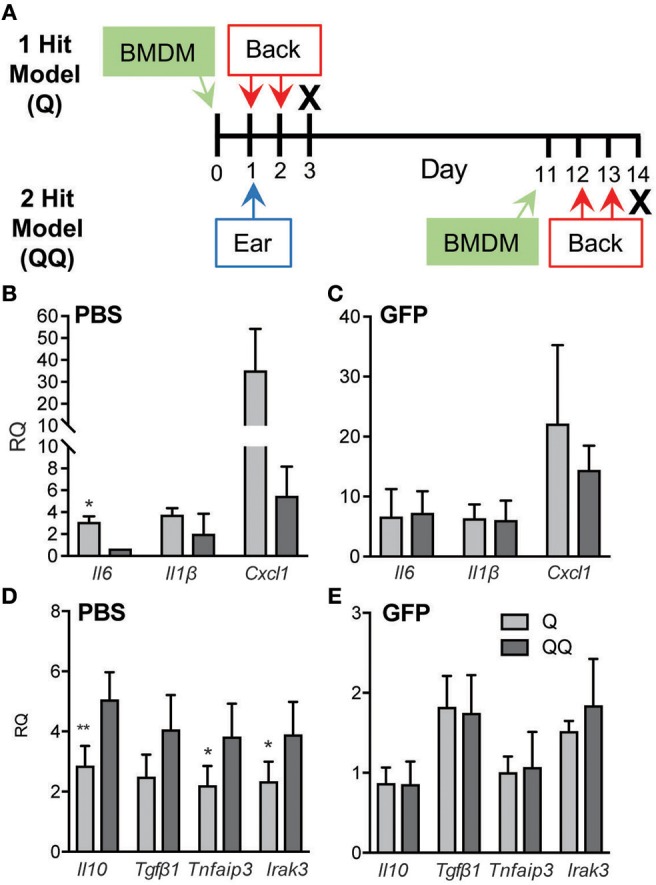
Adoptive transfer of naïve macrophages blocked tolerance. **(A)** Schematic diagram of adoptive transfer protocol. BMDM (7 × 10^6^ cells/recipient) from wildtype GFP mice **(B,D)** or an equal volume of PBS **(C,E)** were administered to wildtype mice 1 day before 2-day IMQ applications to the back in the 1- and 2-hit protocols. Back skin was collected for analysis of *Il6, Il1b*, and *Cxcl1* mRNAs using qPCR. Data are normalized to *Hprt* and *Gapdh* and shown as fold change relative to mice receiving BMDM but no IMQ treatment. Data are from two independent experiments, with *n* ≥ 3 in each experiment. (^*^p ≤ 0.05, ^**^p ≤ 0.01).

To further test the role of macrophages, we applied IMQ to the ears of wildtype mice, then adoptively transferred naïve wildtype GFP^+^ BMDM 1 day before the back-skin applications of IMQ in both the 1- and 2-hit models (Figure 5A). Administration of naïve macrophages had no impact on the immune responses to 1-hit. Evidence of tolerance was seen in control mice (Figures 5B,D; Q vs. QQ, PBS group); however, adoptive transfer of naïve macrophages abrogated the development of tolerance in the 2-hit model as gauged by the elevated levels of *Il6, Il1b*, and *Ccxl1* and suppression of *Il10, Tgf*β*1, TNFAIP3* and *IRAK3* mRNAs (Figures 5C,E; BMDM groups). These findings indicate that macrophages are critical for the development of tolerance to TLR7 activation.

### MMP10 Mediates Tolerance to IMQ in Macrophages

**Figure 6:**
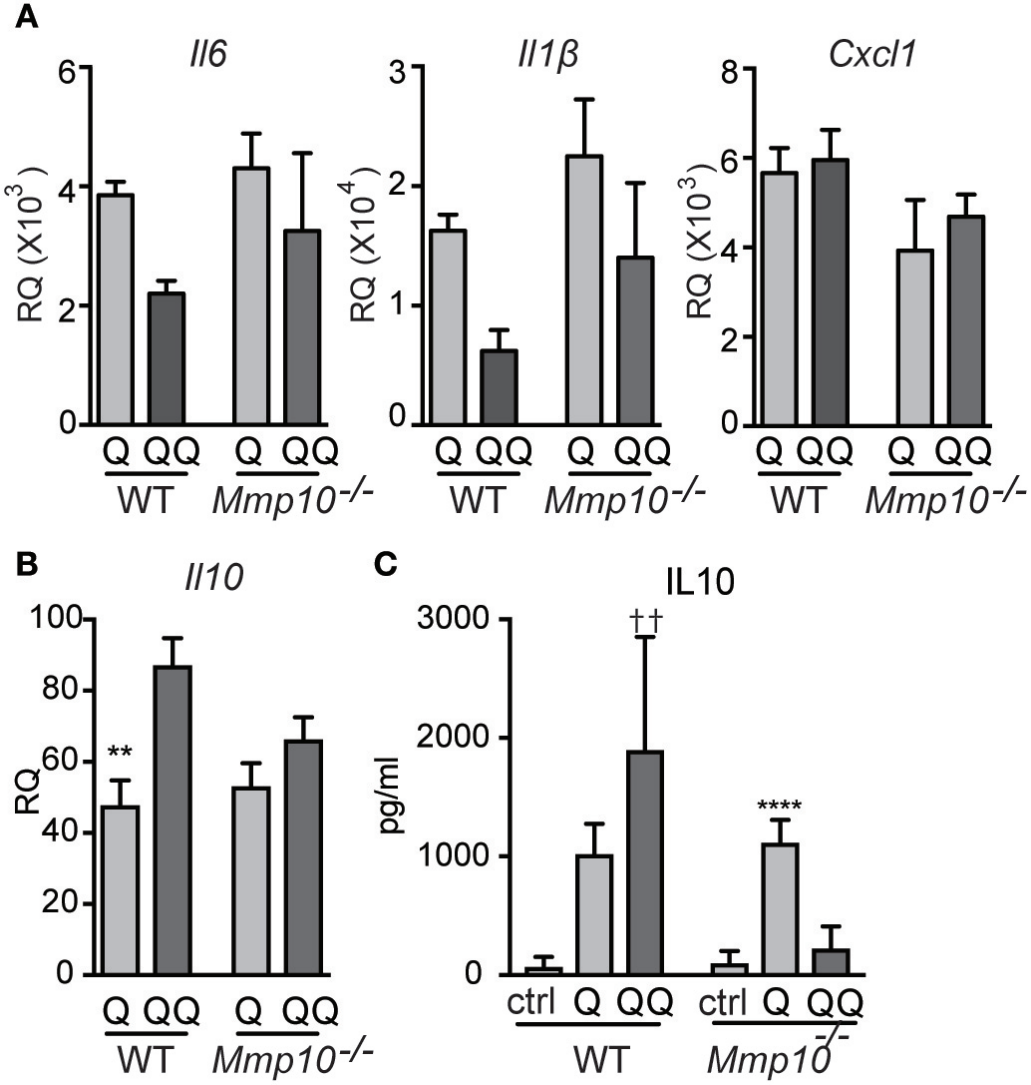
Hypo-responsiveness of macrophages in culture to repeated TLR7 stimulation is mediated by MMP10. Wildtype and *Mmp10*^−/−^ BMDM were stimulated with IMQ for the 1-hit (Q) or 2-hit model. **(A,B)** mRNA expression levels were quantified by qPCR and normalized to *Hprt*. Data are normalized to unstimulated wildtype control cells. **(C)** IL10 was measured in supernatant from cells using ELISA, data are representative of 3 individual experiments (*n* = 3). (^**^p ≤ 0.01: WT-Q vs WT-QQ and ^****^p ≤ 0.0001: *Mmp10*^−/−^Q vs. *Mmp10*^−/−^QQ;*p* ≤ 0.01: WT-QQ vs *Mmp10*^−/−^QQ).

Pre-sensitized macrophages are tolerant to subsequent stimulation to TLR7/8 ligands (31). Our published *in vivo* data in other models indicate that MMP10 regulates macrophage function and activation status (13, 14), and our data here indicates that this proteinase also controls hypo-responsiveness to TLR7 signaling. As MMP10 is primarily a product of macrophages, we assessed if macrophage MMP10 affects tolerance responses in isolated cells. We treated wildtype and *Mmp10*^−/−^ BMDM with IMQ (1-hit and 2-hit models, exposures modified as described under Methods). As evident by reduced expression of *Il6* and *Il1b* in the 2-hit model, we found that wildtype macrophages were hypo-responsive to subsequent stimulation by IMQ whereas *Mmp10*^−/−^ macrophage were not (Figure 6A), just as we saw *in vivo* (Figure 1B). Furthermore, and as we found *in vivo*, tolerized wildtype macrophages (QQ), expressed higher levels of IL10 (Figures 6B,C). We found no difference in expression of between *Tnfaip3* between wildtype and *Mmp10*^−/−^ macrophages, and decreased expression of IRAK3 and TGFβ1 in 2-hit vs. 1-hit treatment in *Mmp10*^−/−^ BMDM, but no change in wildtype BMDM (Figure S7A). In addition, we found no difference in TLR7 expression between wildtype and *Mmp10*^−/−^ macrophages (Figure S7B), suggesting that MMP10 impacts tolerance downstream of TLR7 signaling.

## Discussion

In this study, we demonstrated that topical application of IMQ, a TLR7 agonist, induced systemic tolerance and dampened the inflammatory responses to subsequent application of this ligand. The hypo-responsiveness in both the expression of pro-inflammatory cytokines and neutrophil influx was associated with increased expression of anti-inflammatory cytokines IL10 and TGFβ1 and the ubiquitin editing enzyme TNFAIP3, which negatively regulates NFκB signaling (32). We found that macrophages were essential effector cells in mediating these responses and that MMP10, a macrophage product, was required for tolerance to develop.

Plasticity and diversity are hallmarks of macrophages, and a range of signaling molecules can modulate their state of activation (33). TLR engagement typically drives macrophage polarization toward pro-inflammatory phenotype (classically-activated macrophages or M1), whereas tolerance seems to be related to alternatively-activated or M2 macrophages (34–37). We reported that Mmp10 drives the conversion of macrophages from pro-inflammatory, M1-biased cells to an anti-inflammatory, M2-biased cells (14). Although we do not yet understand the mechanism of its action, because several *Mmp10*^−/−^ phenotypes seen *in vivo* are duplicated with macrophages in culture (13, 14), we predict that MMP10 cleaves a protein on the surface of M1-polarized macrophages that either by gain- or loss-of-function promote the transition to an M2-biased state. A focus of our lab's effort is to identify and validate the MMP10 substrate that controls macrophage activation.

In agreement with findings from others (21, 22), our *in vivo* and *in vitro* data support an important role for macrophages in tolerance to TLR7 ligation. Similar to our findings with *Rag1*^−/−^ mice, Hayashi et al. (17) reported that tolerance to 1V136, another TLR7 agonist, does not require T and B cells. However, Bourquin et al. (18) reported that pre-exposure of plasmacytoid and myeloid dendritic cells to Resiquimod, an analog of IMQ, blocks release of IL6, IL12p70, and IFN-α in response to a subsequent exposure, suggesting that these leukocytes also play a role in mediating tolerance *in vivo*. As we determined that dendritic cells do not express MMP10 (14), the MMP10-dependent function in tolerance would be limited to macrophages. Furthermore, our adoptive transfer studies underscore a critical role for macrophages in mediating tolerance to TLR7 ligation.

Although keratinocytes express MMP10 in response to injury and produce inflammatory markers, such as TNFα and IL8 (38, 39), we do not believe that these cells have a major role in mediating tolerance to TLR7 ligation. Compared to macrophages, these epidermal cells are much less sensitive to TLR7 activation, likely because they do not express TLR7 at meaningful levels. High concentrations of IMQ (100 μm) are needed to induce keratinocyte activation (39). Furthermore, using a IMQ model of psoriasis (40), we found no difference in epithelial thickness between wildtype and *Mmp10*^−/−^ mice (unpublished data).

Compared to their TLR7-tolerance model, in which systemic administration leads to tolerance within 24 h after stimulation and then dissipating within 5 days (18), our cutaneous-application method required a longer interval for hypo-responsiveness to become apparent. Although the sequence of events that block the function of inflammatory macrophages is not fully understood, we speculate that topical application on the ear induced a systemic response and reprogramming of macrophages to an anti-inflammatory state that made the host tolerant of a second hit. Thus, adoptive transfer of naïve macrophages into pre-treated mice overrode the immunosuppressive activity of tolerant monocytes making the response to a second administration of IMQ appear like that seen in the 1-hit model (Figure 6).

The systemic responses to topical application of Aldara cream have been reported (41). Similarly, in our studies, we observed weight loss as indication of systemic responses. Although the 2-hit regimen induced systemic tolerance in *Rag*^−/−^ mice, as evidenced by significantly less weight loss compared to 1-hit model, wildtype mice had a similar weight loss in response to both 1-hit and 2-hit treatment (Figure S1). We speculate that in wildtype mice lymphocytes alleviate systemic inflammation in 1-hit model, thereby obscuring differences in weight loss between the two regimens as was observed in Rag^−/−^ mice.

Matrix metalloproteinase modulate a wide range of immune functions and responses (11, 42, 43). For example, MMP7, MMP8, MMP10, and MMP28 serve beneficial functions in response to acute infection or injury by moderating pro-inflammatory responses (10, 12, 14, 25, 44, 45). Here, we found that macrophage MMP10 was needed to induce tolerance to TLR7 signaling. Regulation of macrophage immune tolerance by MMP10 is a novel function for this proteinase (or for any metalloproteinases), but yet we do not know how MMP10 mediates this regulation. As stated, to understand such mechanisms, we need to identify the protein substrate(s) cleaved by macrophage MMP10, which is the focus of our ongoing studies.

## Ethics Statement

This study was carried out in accordance with the recommendations of Institutional Animal Care and Use Committee at Cedars-Sinai Medical Center. The protocol was approved by the Institutional Animal Care and Use Committee at Cedars-Sinai Medical Center.

## Author Contributions

MR, CJ, and WP conceived and designed the research. MR, ED, AB, and YW performed experiments. MR, CJ, and WP analyzed and interpreted data. MR and WP wrote the manuscript and prepared figures. All authors edited and approved final manuscript.

### Conflict of Interest Statement

The authors declare that the research was conducted in the absence of any commercial or financial relationships that could be construed as a potential conflict of interest.
