# Supplementary material for: Macrophage MMP10 Regulates TLR7-Mediated Tolerance
Source: Front Immunol. 2018 Dec 4;9:2817. doi: 10.3389/fimmu.2018.02817 (PMC6288447; doi:10.3389/fimmu.2018.02817)
Supplement: Supplementary file 1 [file Data_Sheet_1.docx]

**Supplement to**

**Macrophage MMP10 Regulates Tolerance to TLR7 Signaling**

Maryam G. Rohani, Elizabeth Dimitrova, Andrew Beppu, Ying Wang,
Caroline A. Jefferies, and William C. Parks

**
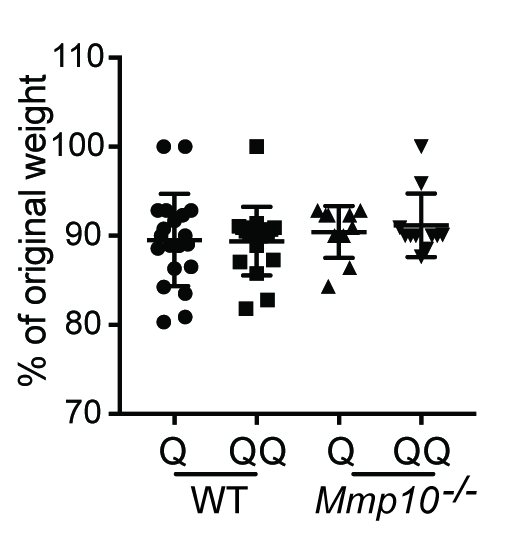
**

**Fig. S1. Wildtype (WT) and *Mmp10^–/–^* mice had similar weight loss in response to 1-hit (Q) and 2-hit (QQ) IMQ treatment.** Data shown are mean ± SEM (each symbol represent one mouse, n ≥ 10).

**
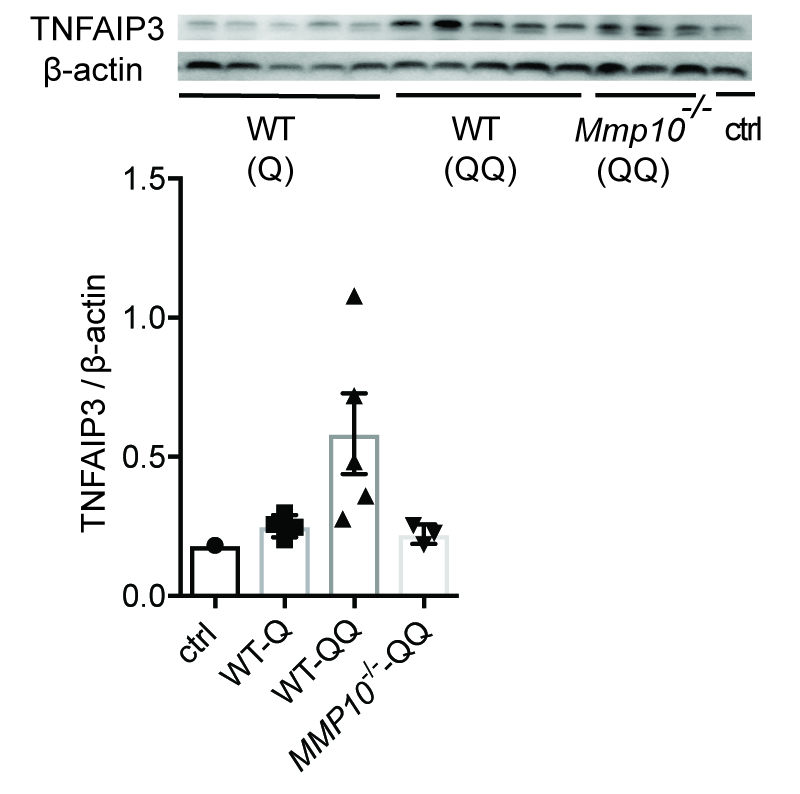
**

**Fig S2. MMP10-dependent expression of TNFAIP3 protein in response to 2-hit treatment.** Skin samples from wildtype (WT) and *Mmp10^–/–^* mice treated with IMQ (1-hit/Q or 2-hit/QQ) were homogenized in PBS. 20 µg of total protein were solubilized in 1X NuPage LDS sample and reducing buffer and resolved by SDS-PAGE. Proteins were electrophoretically transferred to Immobolin^TM^- PVDF membranes. Membranes were incubated with rabbit monoclonal antibody D13H3 (Cell Signaling Technology, Danvers, MA) or goat polyclonal antibody C11 (Santa Cruz Biotechnology Inc, Dallas, TX) overnight at 4 ºC to detect TNFAIP3 and β-actin proteins, respectively. Bound antibodies were visualized using horseradish peroxidase-linked secondary antibodies, followed by detection using SuperSignal West Pico Chemiluminescent substrate (Thermo Scientific, Rockford, IL) according to the manufacturer’s instructions. Band intensities were quantified using ImageJ (NIH, Bethesda, Maryland) and presented as ratio of TNFAIP3/β-actin. Bars show mean ± SEM, and each symbol represents data from one mouse.

**
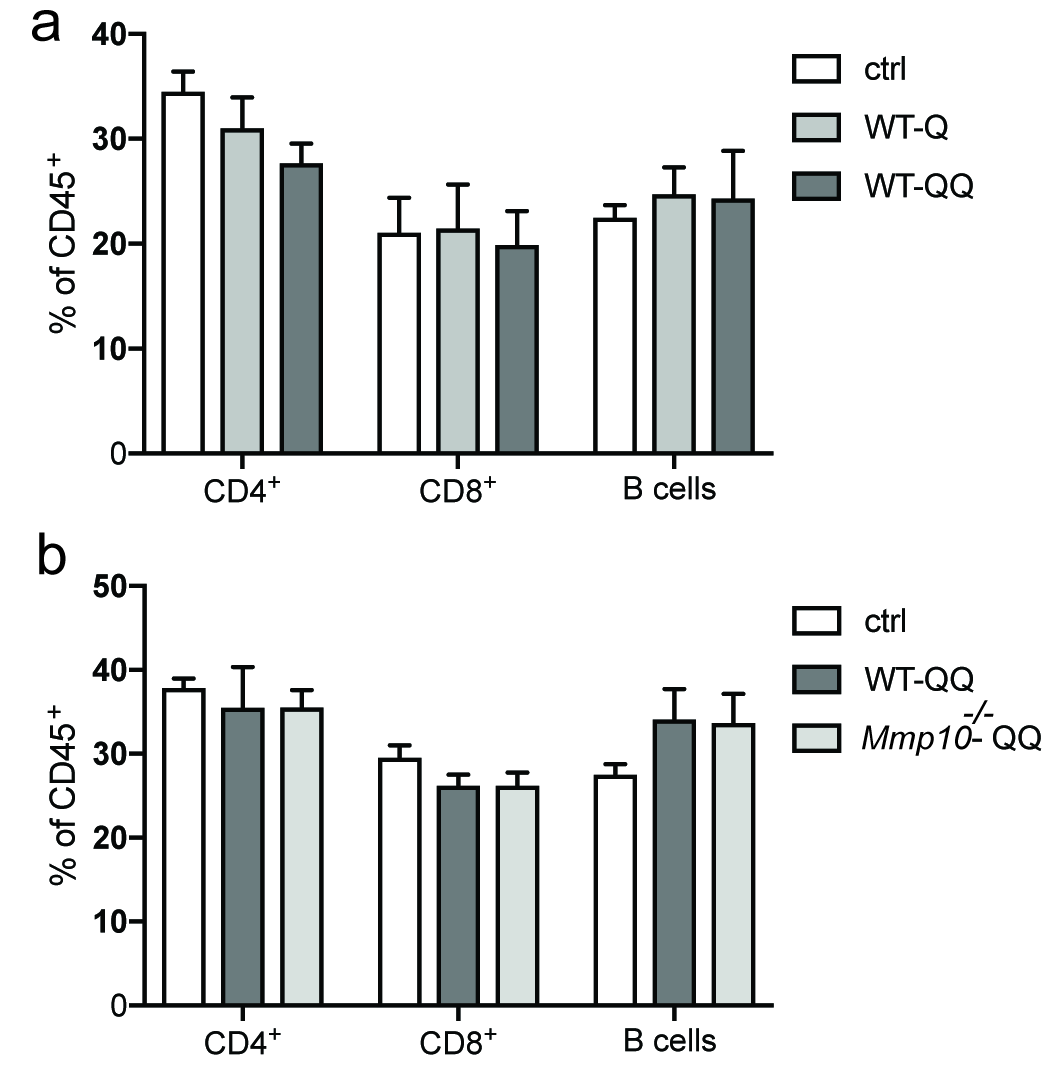
**

**Fig. S3 Lymphocyte populations in lymph nodes were not affected in response to 1-hit and 2-hit IMQ treatments in both wildtype and *Mmp10^–/–^* mice.** Lymph nodes were collected from untreated control (ctrl) mice and treated wildtype (WT) and *Mmp10^–/–^* mice. Single cells were isolated, stained with antibodies against CD45, CD4, CD8, and B220 (see Methods in main text), and analyzed by flow cytometry. CD4^+^ cells were identified as CD45^+^ CD8^-^ CD4^+^, CD8^+^ cells as CD45^+^ CD4^-^ CD8^+^ and B^+^ cells as CD45^+^ CD4^-^ CD8^-^ B220^+^. Data shown are mean ± SEM (n≥ 5).

**
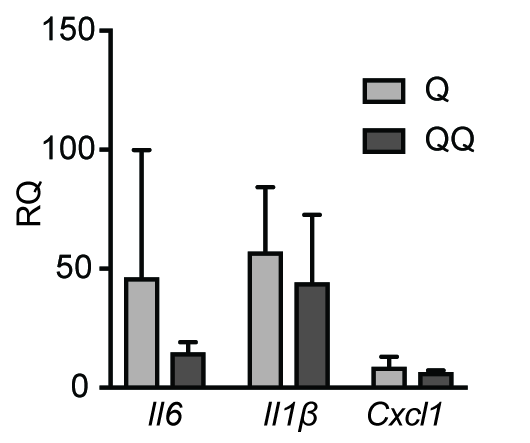
**

**Fig. S4. Expression of pro-inflammatory markers in *Rag^–/–^* mice did not differ between 1-hit/Q and 2-hit/QQ treatments.** *Rag^-/-^* mice were treated with IMQ either by 1-hit or 2-hit treatment as described in the Methods (see Fig. 1a). Back skin was harvested, RNA isolated, and expression of mRNAs for pro-inflammatory markers *Il-6*, *Il-1β,* and *Cxcl1* was assessed by qPCR. Data shown are mean ± SEM (n = 8).

**
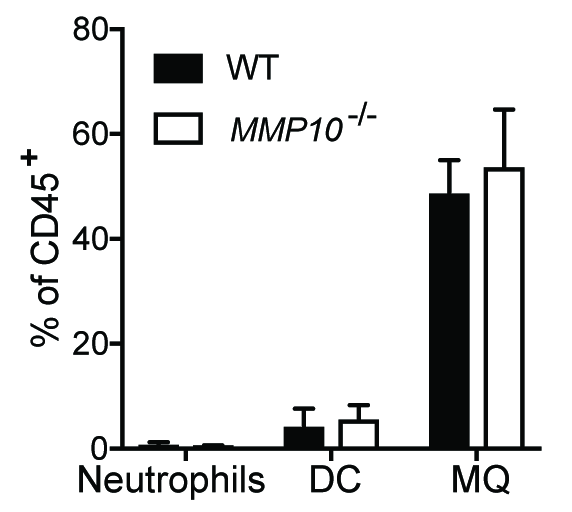
**

**Fig. S5. Before the second hit, the numbers of macrophages, dendritic cells, and neutrophils in back skin did not differ between wildtype and *Mmp10^–/–^* mice.** IMQ (25 mg) was applied to the right ears of wildtype (WT) and *Mmp10^–/–^* mice. On day 12 post-treatment, back skin samples were collected, and cells were isolated, stained with antibodies to CD45, Ly6G, CD11b, F4/80, CD11c, and MHC II, and analyzed by flow cytometry. As shown in the upper diagram, neutrophils were identified as CD45^+^ Ly6G^–^, Dendritic cells as CD45^+^ Ly6G^–^ CD11c^+^ MHC II^+^ and macrophages as CD45^+^ Ly6G^–^ CD11b^+^ F4/80^+^. Data shown are mean ± SEM (n = 3).


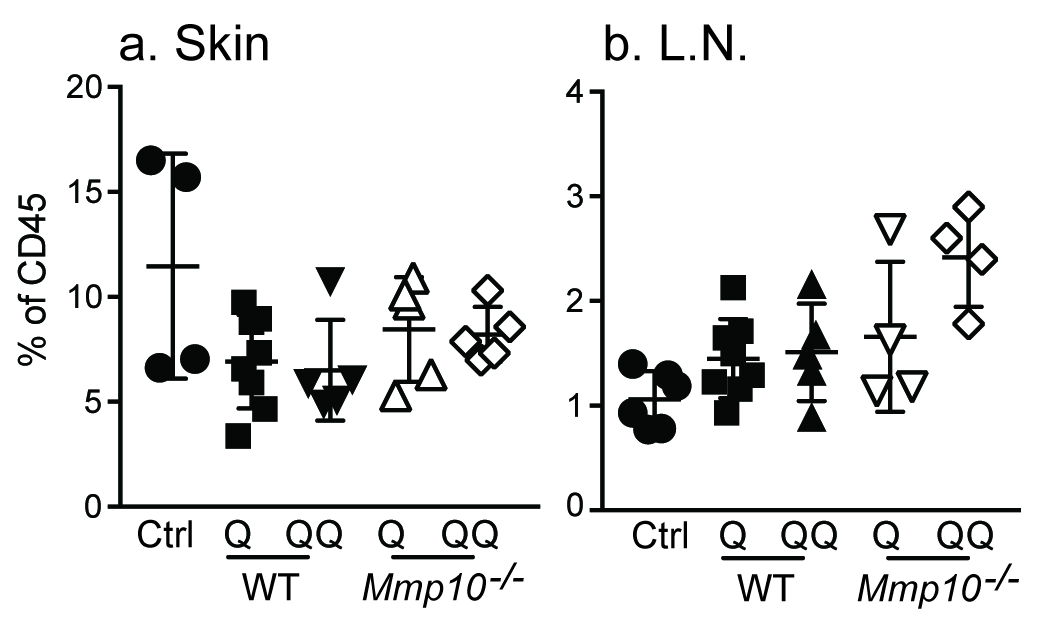


**Fig. S6**. **Dendritic cell numbers in skin and in draining lymph nodes did not differ between 1-hit and 2-hit treatments or between in wildtype (WT) and *Mmp10^–/–^* mice.** Back skin and axillary and inguinal lymph nodes were isolated from untreated control mice (Ctrl) and from wildtype and *Mmp10^–/–^* mice after 1-hit or 2-hit IMQ treatments. Single cells were isolated, stained with antibodies against CD45, Ly6G, CD11c, and MHC II, and analyzed by flow cytometry. Dendritic cells were identified as CD45^+^ Ly6G^–^ CD11c^+^ MHC II^+^. Data shown are mean ± SEM (n≥ 4, each symbol represent data from one mouse).

**
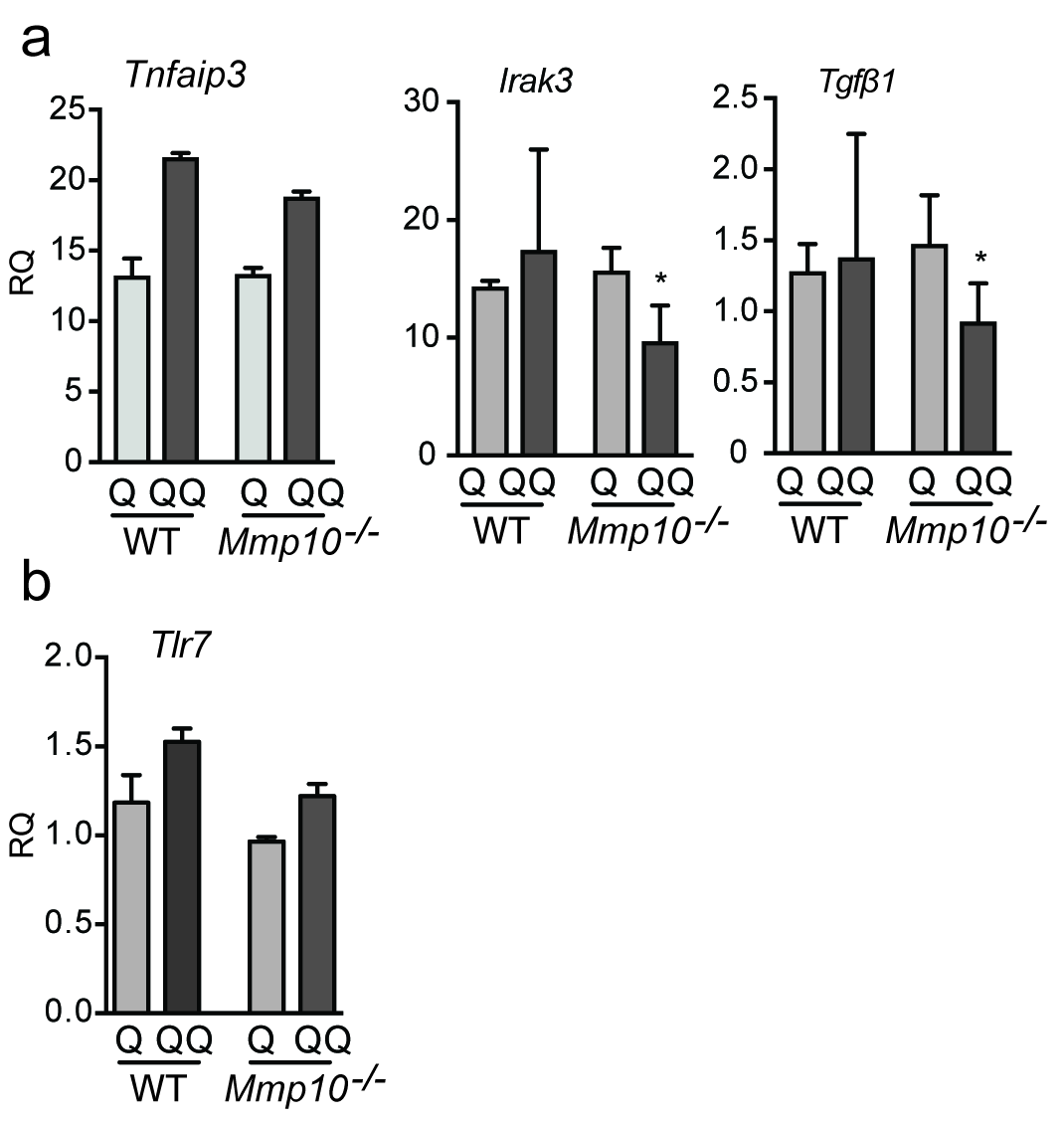
**

**Fig. S7. Responses in cultured macrophages mirror *in vivo* responses 1-hit or 2-hit IMQ treatment.** Wildtype and *Mmp10^–/–^* bone marrow derived macrophages (BMDM) were stimulated with IMQ for the 1-hit (Q) or 2-hit model. mRNA levels were quantified by qPCR and normalized to *Hprt.* Data are normalized to levels in unstimulated wildtype BMDM. data are representative of 3 individual experiments (n=3), p ≤0.05: *WT-Q vs WT-QQ and *Mmp10^–/–^*-Q vs. *Mmp10^–/–^*-QQ.
